# Radiomics features of hippocampal regions in magnetic resonance imaging can differentiate medial temporal lobe epilepsy patients from healthy controls

**DOI:** 10.1038/s41598-020-76283-z

**Published:** 2020-11-11

**Authors:** Yae Won Park, Yun Seo Choi, Song E. Kim, Dongmin Choi, Kyunghwa Han, Hwiyoung Kim, Sung Soo Ahn, Sol-Ah Kim, Hyeon Jin Kim, Seung-Koo Lee, Hyang Woon Lee

**Affiliations:** 1 Department of Radiology and Research Institute of Radiological Science and Center for Clinical Imaging Data Science, Yonsei University College of Medicine, Seoul, Korea; 2 Department of Neurology, Epilepsy and Sleep Center, Ewha Womans University School of Medicine and Ewha Medical Research Institute, 1071, Anyangcheon-ro, Yangcheon-gu, Seoul, 07985 Korea; 3 Department of Medical Science, Ewha Womans University School of Medicine and Ewha Medical Research Institute, Seoul, Korea; 4 Interdisciplinary Programs of Computational Medicine, System Health & Engineering Major in Graduate School, Ewha Womans University, Seoul, Korea; 5 Department of Computer Science, Yonsei University, Seoul, Korea

**Keywords:** Neuroscience, Biomarkers, Medical research, Neurology

## Abstract

To investigative whether radiomics features in bilateral hippocampi from MRI can identify temporal lobe epilepsy (TLE). A total of 131 subjects with MRI (66 TLE patients [35 right and 31 left TLE] and 65 healthy controls [HC]) were allocated to training (n = 90) and test (n = 41) sets. Radiomics features (n = 186) from the bilateral hippocampi were extracted from T1-weighted images. After feature selection, machine learning models were trained. The performance of the classifier was validated in the test set to differentiate TLE from HC and ipsilateral TLE from HC. Identical processes were performed to differentiate right TLE from HC (training set, n = 69; test set; n = 31) and left TLE from HC (training set, n = 66; test set, n = 30). The best-performing model for identifying TLE showed an AUC, accuracy, sensitivity, and specificity of 0.848, 84.8%, 76.2%, and 75.0% in the test set, respectively. The best-performing radiomics models for identifying right TLE and left TLE subgroups showed AUCs of 0.845 and 0.840 in the test set, respectively. In addition, multiple radiomics features significantly correlated with neuropsychological test scores (false discovery rate-corrected *p*-values < 0.05). The radiomics model from hippocampus can be a potential biomarker for identifying TLE.

## Introduction

Temporal lobe epilepsy (TLE) is the most frequent type of focal epilepsy, and also the type which is most refractory to drug treatment^1^. For many of these patients, surgical resection of the epileptic focus offers a viable treatment option to eliminate seizures, and noninvasive imaging plays a pivotal role in precisely identifying the epileptogenic zone^2,3^. One of the histopathologic landmarks of TLE is hippocampal sclerosis, which presents in approximately 54% of surgically treated drug-refractory patients^4,5^. Previous neuroimaging studies on TLE have mainly focused on volumetric analysis, diffusion tensor image (DTI) studies, or functional MRI studies^6–8^. Volumetric analyses revealed abnormalities in the hippocampus ipsilateral to the seizure focus, which is the hallmark imaging finding in TLE^9–11^. However, these studies focused on single parameters such as mean apparent diffusion coefficient, mean fractional anisotropy, or volume rather than focusing on the spatial distribution of heterogeneity. Also, it is known that TLE patients show cognitive impairments, affecting not only memory, but also a broad array of cognitive capacities including executive functions, language, and sensorimotor skills^12–14^. Previous MRI studies have shown structural and functional damage associated with cognitive impairments in TLE patients^12,15–17^, but these studies have also focused on single conventional parameters.

Radiomics is a rapidly developing study field that extracts comprehensive and automated quantifications of radiographic phenotypes using data characterization algorithms^18^. Because radiomics models use high-throughput imaging features, they are able to discover hidden information that is inaccessible when using single-parameter approaches. High-dimensional radiomics data provide insight into intraregional heterogeneity and reflects the spatial complexity of a disease, which are invisible to the human eye. Compared with previous techniques that process medical images for visual examination, radiomics introduces a new method for data mining and thus offers novel opportunity for clinically assisted diagnosis^19^. We hypothesized that radiomics features from routine conventional MRI would result in distinct combinations of imaging parameters that identify TLE. We additionally assessed whether these radiomics features were correlated with cognitive impairments in these TLE patients.

## Methods

### Patient population

Written informed consent was obtained from legally authorized representatives of TLE patients and HCs, and the study protocol was approved by the local Ethical Committee at Ewha Medical Center. All the experiment protocol for involving humans was in accordance to guidelines of national/international/institutional or Declaration of Helsinki. Sixty-six TLE patients who had visited Ewha Womans University Mokdong Hospital for both long-term video encephalographic (EEG) monitoring and MRI scans between July 2013 and August 2018 were enrolled. The clinical diagnosis of TLE was made according to the criteria of the International League Against Epilepsy^20,21^. Based on electroclinical findings, we included TLE patients with recurrent unprovoked seizures, with or without secondary generalization, originating from the temporal lobe.

All patients had unilateral temporal interictal spikes or unilateral temporal lobe seizure onset on EEG. TLE patients were further divided into those with a right-sided (n = 35) or left-sided (n = 31) seizure focus. The seizure focus was determined by predominantly ipsilateral interictal epileptic abnormalities (70% cutoff) and unequivocal seizure onset recorded during prolonged video-EEG monitoring in all patients^22^. Patients were excluded from the study if they had contralateral or extratemporal epileptiform discharges on EEG, previous brain surgery, chronic medical illness with central nervous system involvement other than epilepsy, contraindication for MRI, or history of drug abuse or psychiatric illness other than axis I depressive disorders. For the control group, 65 healthy controls (HCs) (age = 40.5 ± 12.1 years, 30 females) were recruited. The HCs had no history of neurological disorders and no MRI abnormalities. An institutional cohort of 131 subjects (66 TLE patients and 65 HCs) was enrolled in the study.

To differentiate TLE from HCs, the institutional cohort was split into training (n = 90) and test (n = 41) sets, with stratification for TLE presence. Also, because TLE is a lateralized disease, we performed subgroup analyses in (1) right TLE vs. HC, in which right TLE and HC were included and semi-randomly allocated to training (n = 69) and test (n = 31) sets, and (2) left TLE vs. HC, in which left TLE and HC were included and semi-randomly allocated to training (n = 66) and test (n = 30) sets.

Verbal memory function, visual memory function, language function, and frontal executive function were assessed using a Korean version of the California Verbal Learning Test (CVLT)^23^, Rey Complex Figure Test (RCFT)^24^, Korean version of the Boston Naming Test (K-BNT)^25^, sematic Controlled Oral Word Association Test (COWAT) and Stroop test, respectively (detailed information in Supplementary Material S1). The Mini-Mental State Examination (MMSE) was performed in TLE patients only.

### MRI protocols

MRI was performed using a 3.0 T scanner (Philips Achieva v 2.6, Philips, Andover, MA, USA) in all subjects. The MRI included T1-weighted (TR/TE 1160/4.19 ms; field of view 140 × 250 mm, section thickness 1.2 mm, matrix 256 × 192 mm, flip angle 15°), T2-weighted axial/oblique coronal and FLAIR axial/oblique coronal images (5 mm section thickness for each sequence), as well as axial T2* GRE (TR/TE 720/16 ms; field of view 220 × 220 mm, section thickness 5 mm, matrix 256 × 229 mm, flip angle 18°) in TLE patients. HCs underwent identical T1-weighted MRI sequences.

### Image preprocessing and radiomics feature extraction

Preprocessing of the images was performed to standardize the data analysis across patients. We resampled the original images to a 1 mm isovoxel and used FreeSurfer 5.3.0 (surfer.nmr.mgh.harvard.edu) to obtain subject-specific masks of brain regions as defined by the Desikan-Killany atlas^26^. This procedure involved motion correction of T1-weighted images, removal of non-brain tissue^27^, automatic Talairach transformation, segmentation of subcortical white matter and deep gray matter structures^28^, intensity normalization, tessellation of the gray matter/white matter boundary^29^, automated topology correction, and surface deformation following intensity gradients^30^. Once the cortical models were complete, they were registered to a spherical atlas, and the cerebral cortex was parcellated into units with respect to gyral sand sulcal structures^31^. We mapped the brain parcellation mask for each subject from FreeSurfer space to the isovoxel native space and extracted two regions of interest (ROI) masks (right and left hippocampi). We visually checked for segmentation or registration errors by overlaying each subject’s native-space-transformed ROI masks onto their T1-weighted images.

Non-uniform low-frequency intensity was removed by applying the N4 bias correction algorithm. Next, arbitrary T1 signal intensities were normalized using the Whitestripe normalization algorithm^32^. All images were resampled to 1 mm isovoxels across all patients.

The following radiomics features were extracted from each ROI on T1-weighted images using an open-source package (PyRadiomics, version 1.3.0)^33^: (1) 14 shape features, (2) 18 first-order features, and (3) 61 s-order features (including gray level co-occurrence matrix, gray level run-length matrix, gray-level size zone matrix, and neighboring gray tone difference matrix) (detailed information on Supplementary Table S1). A total of 186 radiomics features were extracted (93 features x two ROIs [right and left hippocampus]).

### Statistical analysis

For univariate analysis of baseline characteristics and neuropsychological test scores, either the Student’s t-test or Mann Whitney’s *U* test was used for continuous variables, depending on the normality of the data. Chi-square test was performed for categorical variables. Statistical analysis was performed using the statistical software R (version 3.5.1; R Foundation for Statistical Computing, Vienna, Austria).

### Radiomics feature selection and machine learning models

All imaging features were normalized using z-score normalization. For feature selection, the F-score, least absolute shrinkage and selection operator (LASSO), and mutual information (MI) with ten-fold cross validation was applied. After feature selection, the radiomics classifiers were constructed by machine learning models including support vector machine (SVM), logistic regression (LR), or AdaBoost, with ten-fold cross-validation.

Hyperparameters were optimized by random search. Thus, a total of 9 combinations of machine learning algorithms were trained and validated. Performance was evaluated in the training sets and validated in the test sets to differentiate the entire TLE group from HC group, without controlling the alignment of the epileptic side. Further analysis was performed to differentiate the ipsilateral TLE group from the HC group. In subgroup analyses of (1) right TLE vs. HC and (2) left TLE vs. HC, feature selection and machine learning were performed separately. In addition, to overcome data imbalances in subgroup analyses, each machine learning model was trained (1) without over-sampling, or (2) with SMOTE (synthetic minority over-sampling technique)^34,35^. Thus, a total of 18 combinations of machine learning algorithms and over-sampling were trained and validated for each subgroup. Performance was evaluated in the training set and validated in the test set. Area under the curve (AUC), accuracy, sensitivity, and specificity were estimated.

Based on the radiomics classification model in the training set, the best combination of feature selection, classification methods, and subsampling in each model was used in the test set.

**Figure 1:**
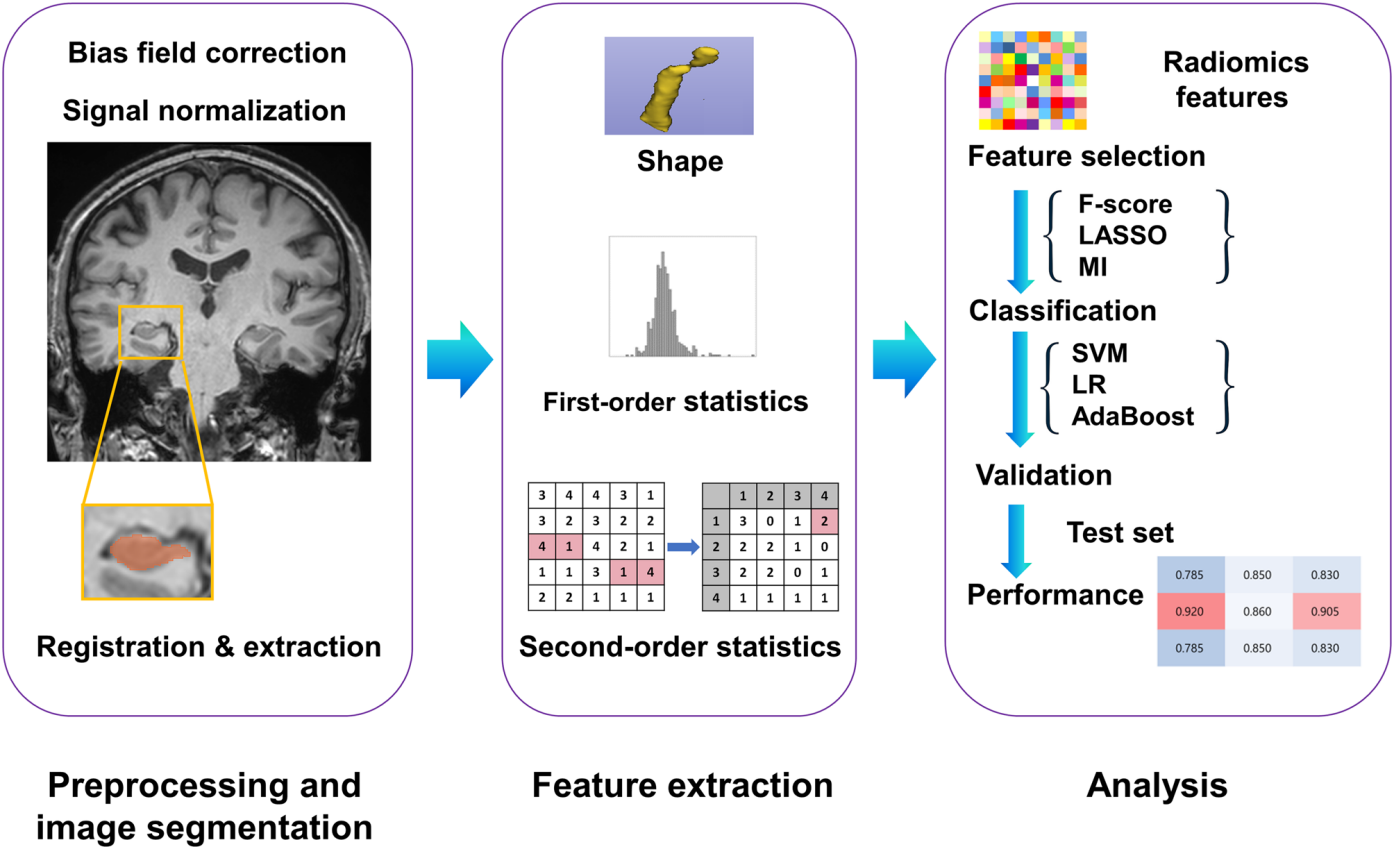
Workflow of image processing, radiomics feature extraction, and machine learning.

The different feature selection, over-sampling, and classification methods computed using Machine learning were performed using Python 3 with Scikit-Learn library v 0.21.2. The threshold for statistical significance was set at* p* < 0.05. The overall process is shown in Fig. 1.

### Comparison of the diagnostic performance with that of human readers

We tested the diagnostic performance to differentiate TLE from HC in training and test set on T1-weighted images by a consensus of 2 readers (a neuroradiologist with 9 years of experience and a neurologist with 27 years of experience, respectively), who were blinded to the clinical information. The performance of radiomics model and human readers were compared using AUC with DeLong’s method^36^.

### Correlations between radiomics features and neurocognitive function

Pearson’s correlation coefficient was calculated to evaluate the relationships between the identified radiomics features and significantly different neuropsychological results between TLE and HCs (FDR-corrected *p* < 0.05).

## Results

### Subject characteristics

The baseline characteristics and neuropsychological test results of TLE patients and HCs are summarized in Supplementary Table S2. TLE patients showed significant cognitive decrements in all cognitive domains (including language, verbal memory, visual memory, and frontal/executive function), compared with HCs. Left TLE patients showed worse language functions than right TLE patients on K-BNT scores (32.9 vs. 58.0, *p* = 0.002). Otherwise, there were no significant differences in verbal memory, visual memory, and frontal/executive functions between right TLE and left TLE patients (Supplementary Table S3).

The clinical characteristics of the subjects in the training set and test set are summarized in Table 1. There were no differences in the clinical characteristics between the training and test sets.

**Table 1:** Clinical characteristics in the training and test sets.

	Training set (n = 90)	Test set (n = 41)	*p* value*
**TLE vs HC**			
Age (years)	41.8 ± 11.4	40.8 ± 13.6	0.668
Sex (female)	46 (51.1)	23 (56.1)	0.596
Subjects no. (%)			0.989
HC	45 (50)	20 (48.8)	
Right TLE	24 (26.7)	11 (26.8)	
Left TLE	21 (23.3)	10 (24.4)	

	Training set (n = 69)	Test set (n = 31)	*p* value*
**Right TLE vs HC**			
Age (years)	41.0 ± 11.6	42.5 ± 12.8	0.587
Sex (female)	33 (47.8)	18 (58.1)	0.344
Subjects no. (%)			0.946
HC	45 (65.2)	20 (64.5)	
Right TLE	24 (34.8)	11 (35.5)	

	Training set (n = 66)	Test set (n = 30)	*p* value*
**Left TLE vs HC**			
Age (years)	41.6 ± 11.8	39.3 ± 13.3	0.393
Sex (female)	31 (47)	17 (56.7)	0.378
Subjects no. (%)			0.883
HC	45 (68.2)	20 (66.7)	
Left TLE	21 (31.8)	10 (33.3)	

Data are number of subjects. Numbers in parentheses are percentages.

*HC* healthy control, *TLE* temporal lobe epilepsy.

*p values were calculated using Student’s t-test for continuous variables and Chi-square test for categorical variables, to compare subject characteristics of the training and test set.

### Radiomics features and classification performance

#### Entire TLE vs. HC

**Figure 2:**
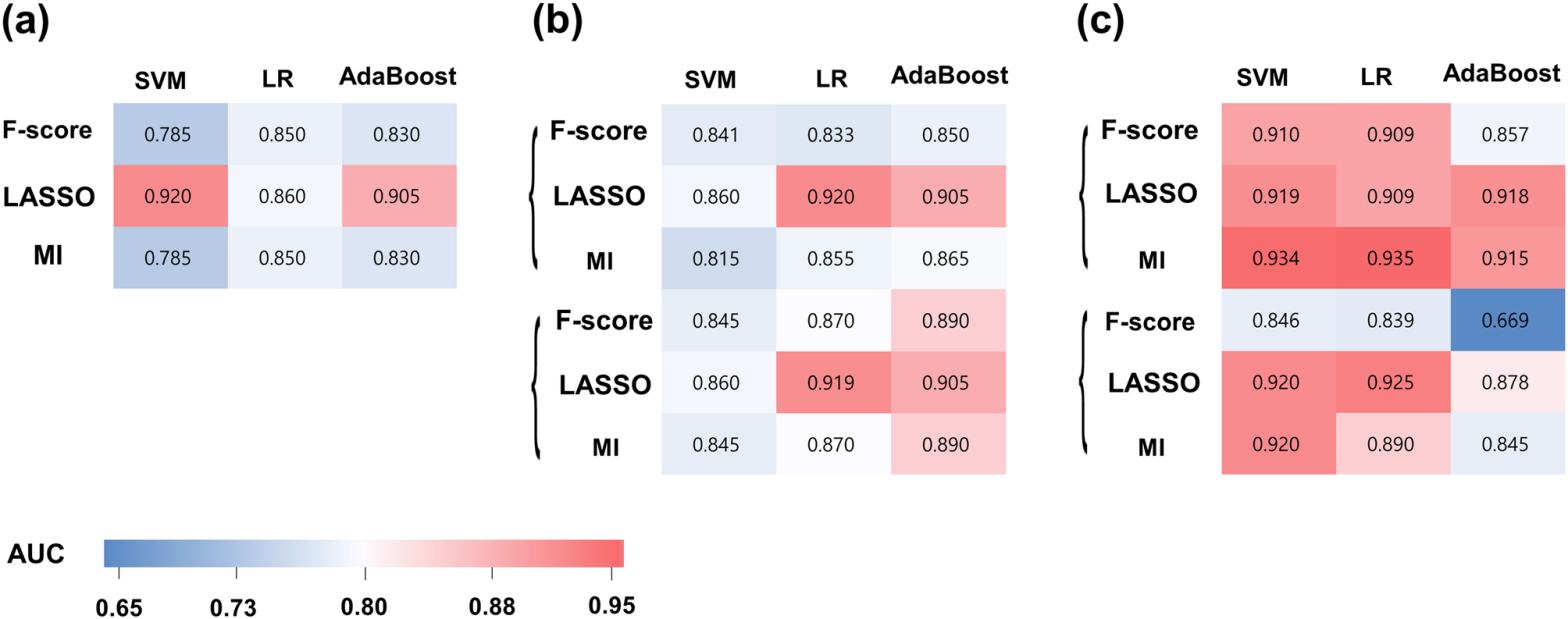
Heatmap of AUC values achieved from the machine learning classifiers in the training sets for (**a**) differentiating TLE from HC, (**b**) differentiating right TLE from HC, and (**c**) differentiating left TLE from HC. *AUC* area under the curve, *MI* mutual information, *LASSO* least absolute shrinkage and selection operator, *LR* logistic regression, *MI* mutual information, *SMOTE* synthetic minority over-sampling technique, *SVM* support vector machine.

Table 2 summarizes the results of the best performing models in the training and test sets. The performance of the 9 combination of models in the training set is shown in Fig. 2a. In the training set, the AUCs of the models ranged from 0.680 to 0.920. LASSO feature selection and SVM showed the best diagnostic performance in the training set, with an AUC, accuracy, sensitivity, and specificity of 0.920 (95% confidence interval [CI] 0.870–0.970), 80.5%, 85.7%, and 80%, respectively. The selected features (10 from right and 6 from left hippocampus) consisted of 2 first-order features, 10 s-order features, and 4 shape features (detailed information in Supplementary Table S4). In the test set, the LASSO feature selection with SVM showed an AUC, accuracy, sensitivity, and specificity of 0.848 (95% CI 0.731–0.964), 84.8%, 76.2%, and 75%, respectively.

**Table 2:** Diagnostic performance of the best performing machine learning model in the training set and the test set.

Feature selection	No. of features	Classification + subsampling	Training set				Test set			
			AUC (95% CI)	Accuracy (%)	Sensitivity (%)	Specificity (%)	AUC (95% CI)	Accuracy (%)	Sensitivity (%)	Specificity (%)
**TLE vs HC**										
LASSO	16	SVM + none	0.920 (0.870–0.970)	80.5	85.7	80	0.848 (0.731–0.964)	84.8	76.2	75
**Right TLE vs HC**										
F-score	30	LR + SMOTE	0.920 (0.870–0.970)	81.1	94	68.5	0.845 (0.723–0.968)	77.4	72.7	80
**Left TLE vs HC**										
LASSO	18	LR + none	0.935 (0.893–0.977)	87.8	82.5	93	0.840 (0.699–0.981)	73.3	70	75

*AUC* area under the curve, *CI* confidence interval, *HC* healthy control, *LASSO* least absolute shrinkage and selection operator, *LR* logistic regression, *MI* mutual information, *SMOTE* synthetic minority over-sampling technique, *SVM* support vector machine, *TLE* temporal lobe epilepsy.

#### Right TLE vs. HC

The performance of the 18 combination of models in the training set is shown in Fig. 2b. In the training set, the AUCs of the models ranged from 0.734 to 0.891. F-score feature selection, with LR and SMOTE showed the best diagnostic performance in the training set, with an AUC, accuracy, sensitivity, and specificity of 0.920 (95% CI 0.870–0.970), 81.1%, 95%, and 68.5% in the test set, respectively.

The selected features (14 from right and 16 from left hippocampus) included 23 first-order features, 3 s-order features, and 4 shape features. In the test set, the F-score feature selection with LR and SMOTE showed an AUC, accuracy, sensitivity, and specificity of 0.845 (95% CI 0.723–0.968), 77.4%, 72.7%, and 80%, respectively.

#### Left TLE vs. HC

The performance of the 18 combination of models in the training set is shown in Fig. 2c. In the training set, the AUCs of the models ranged from 0.845 to 0.935. LASSO feature selection, LR, with SMOTE showed the best diagnostic performance in the training set, with an AUC, accuracy, sensitivity, and specificity of 0.935 (95% CI 0.893–0.977), 87.8%, 982.5%, and 93% in the test set, respectively.

The selected features (5 from right and 13 from left hippocampus) included 2 first-order features, 12 s-order features, and 5 shape features. In the test set, the LASSO feature selection with SVM showed an AUC, accuracy, sensitivity, and specificity of 0.840 (95% CI 0.699–0.981), 73.3%, 70%, and 75%, respectively.

### Comparison of the diagnostic performance of human readers

The reviewers correctly identified 14 and 7 cases of TLE in the training and test sets. The AUC, accuracy, sensitivity, and specificity was 0.617 (0.452–0.764), 17.1%, 23.3%, and 100% in the test set, respectively. The radiomics model showed significantly better performance than human readers did (*p*-value = 0.001).

### Correlations between radiomics features and neuropsychological scores

Among the 16 significant radiomics features from LASSO in differentiating TLE from HC, three were significantly correlated with K-BNT score, two were significantly correlated with CVLT direct recall score, and one was significantly correlated with RCFT immediate recall score (all FDR-corrected *p*-values < 0.05) (Table 3).

**Table 3:** Summary of radiomics features showing significant correlation with neuropsychological test results.

Radiomics features	Pearson’s correlation coefficients	FDR-corrected *p*-value
**K-BNT**		
right_T1_GLSZM_SmallAreaLowGrayLevelEmphasis	− 0.303	0.022
Left_T1_shape_MeshVolume	0.378	0.004
Left_T1_shape_Maximum2DDiameterColumn	0.296	0.023
**CVLT-direct recall**		
Left_T1_shape_Maximum2DDiameterRow	0.230	0.003
Left_T1_shape_MeshVolume	0.241	0.016
**RCFT-direct recall**		
Left_T1_shape_MeshVolume	0.308	0.020

*CVLT* California verbal learning test, *FDR* false discovery rate, *GLSZM* gray level size zone matrix, *K-BNT* Korean version of the Boston Naming Test, *RCFT* Rey complex figure test.

## Discussion

In our study, radiomics analysis of hippocampus showed promising results, with an AUC of 0.848, 0.845, and 0.840 for identifying the entire TLE as well as right TLE and left TLE in the test set, respectively. Surgical resection of the epileptogenic zone, the region which is necessary and sufficient for the initiation of seizures, is an additional treatment option which can achieve seizure freedom^37^. Our radiomics approach with machine learning may be applicable to surgical planning by early identification, and thus allow for an improved patient selection and counseling. Our radiomics model was particularly useful in showing better diagnostic performance than visual inspection of human readers, which is the current standard approach, showing its utility. Previous radiomics studies in the neuroradiology field have mostly focused on brain tumors^38–42^. Our study shows that a radiomics model can be a useful biomarker for identifying TLE.

Notably, our model included both right and left TLE in the entire TLE dataset. TLE is considered to display a strong asymmetrical distribution of abnormalities such as volume loss or white matter abnormalities, primarily observed ipsilateral to the seizure onset site^7^. However, it is also known that the contralateral side of seizure onset may also show volume loss or white matter alterations, although less prominently than the ipsilateral side^7,17,43,44^. Our radiomics model showed a good performance (AUC 0.848) in differentiating the entire TLE from HC, indicating that radiomics has the potential for creating a generalized model that is not influenced by the laterality of TLE. Our results are in agreement with previous studies reporting that microstructural changes precede macroscopic atrophy^45^, and that radiomics may reflect microstructural information different from that provided by volumetric measures.

Neuropathological research has revealed various patterns of neuronal cell loss or gliotic changes within the hippocampus, including hippocampal sclerosis, which is the most common histopathologic abnormality^46,47^. MRI T1 relaxation time is a direct reflection of tissue characteristics and has been reported to independently predict histological measures of neuronal density in TLE^48^. Such variations in relaxation time, which directly cause variations in MRI signal intensity, may provide information beyond that provided by volumetric measures. Radiomics features, especially second-order features, capture the spatial variation in T1 signal intensity that may reflect the underlying pathophysiology, which may explain our observation.

In our study, we have applied domain knowledge in performing radiomics analysis in bilateral hippocampi, which is the key structure involved in TLE^7^. However, because radiomics features tend to extract agnostic information that is invisible to human eyes, one cannot assume which feature will be most important in identifying TLE. Thus, we have further narrowed down the significant features by using various feature selection methods. This methodology results in creating a more generalized and stable classifier that is robust against the idiosyncrasies of the training data^49,50^.

Further, it is known that TLE patients show cognitive impairments, affecting not only memory, but also a broad array of cognitive capacities including executive functions, language and sensorimotor skills^12–14^. Previous MRI studies have shown structural and functional compromises associated with cognitive impairment in TLE patients^12,15–17^, but these studies were also focused on single conventional parameters. Other studies have shown dysfunctional networks related to cognitive impairment, seizure severity, or seizure related change in TLE patients in task-based and/or resting-state functional MRI studies^8,50–55^. In our study, significant correlations were found between radiomics features and neuropsychological test scores, suggesting that radiomics features can also serve as imaging biomarkers for cognitive capacity in TLE patients.

Our study has several limitations. First, this is a retrospective study in a single institution with a relatively small sample size. Further studies with a larger dataset and external validation are warranted for better assessment. Second, because only the hippocampus masks were included, other important regions such as the amygdala and parahippocampus should be investigated in future studies. Third, T2-weighted or FLAIR images were not included in this study because the MRI protocol for healthy controls did not include the aforementioned sequences. Further studies including radiomics features from T2-weighted or FLAIR images should be performed. Fourth, we preserved the laterality of TLE rather than flipping (right to left or left to right TLE) to evaluate the radiomics model. However, flipping would have allowed using sampling algorithms sparingly. We have performed this method because previous studies have shown that right and left TLE demonstrate asymmetrical and different qualities of hippocampal injuries^53–58^.

In conclusion, radiomics model from the hippocampus can be a potential biomarker for identifying TLE.

## Supplementary information

Supplementary Information

## Data Availability

Our anonymized data can be obtained by any qualified investigators for the purposes of replicating procedures and results, after ethics clearance and approval by all authors.

## Code availability

The code for machine learning was modified from our previous available radiomics analysis package (https://github.com/ChoiDM/YBIGTA_AI_HeLP).
