# Supplementary material for: Radiomics features of hippocampal regions in magnetic resonance imaging can differentiate medial temporal lobe epilepsy patients from healthy controls
Source: Sci Rep. 2020 Nov 11;10:19567. doi: 10.1038/s41598-020-76283-z (PMC7658973; doi:10.1038/s41598-020-76283-z)
Supplement: Supplementary file 1 — Supplementary Information [file 41598_2020_76283_MOESM1_ESM.docx]

**Supplementary Data**

**S1. Verbal memory function, visual memory function, language function, and frontal executive function assessment**

Verbal memory function was assessed using a Korean version of the California Verbal Learning Test (CVLT; immediate recall, 20-minute delayed recall, and recognition). ^1^ The total score over 5 learning trials was calculated as the sum of individual task scores and then adjusted for age and education level. Visual memory function was assessed using the Rey Complex Figure Test (RCFT; immediate recall, 20-minute delayed recall, and recognition) ^2^. Language function was assessed using a Korean version of the Boston Naming Test (K-BNT) ^3^ to measure word retrieval. A total score for 60 items was adjusted for age and education level. Frontal executive function was measured by the sematic Controlled Oral Word Association Test (COWAT) and Stroop test. The Mini-Mental State Examination (MMSE) was performed only in the TLE patients.

Supplementary Table 1. Radiomics features extracted for machine learning

| Feature category | Feature list |
| --- | --- |
| Shape (n = 14) | Volume, surface area, surface area to volume ratio, sphericity, maximum 3D diameter, maximum 2D diameter (column), maximum 2D diameter (row), maximum 2D diameter (slice), major axis, minor axis, least axis, elongation, flatness, mesh volume |
| First-order statistics (n = 18) | Energy, total energy, entropy, minimum, 10^th^ percentile, 90^th^ percentile, maximum, mean, median, interquartile range, range, mean absolute deviation, robust mean absolute deviation, root mean squared, skewness, kurtosis, variance, uniformity |
| GLCM (n = 24) | Autocorrelation, cluster prominence, cluster shade, cluster tendency, contrast, correlation, difference average, difference entropy, difference variance, inverse difference, inverse difference moment, inverse difference moment normalized, inverse difference normalized, informal measure of correlation 1, informal measure of correlation 2, inverse variance, joint average, joint energy, joint entropy, maximal correlation coefficient, maximum probability, sum average, sum entropy, sum of squares |
| GLRLM (n = 16) | Short-run emphasis, long-run emphasis, gray level nonuniformity, gray level nonuniformity normalized, run-length nonuniformity, run-length nonuniformity normalized, run percentage, gray level variance, run variance, run entropy, low gray level run emphasis, high gray level run emphasis, short-run low gray level emphasis, short-run high gray level emphasis, long-run low gray level emphasis, long-run high-gray level emphasis |
| GLSZM (n = 16) | Small area emphasis, large area emphasis, gray level non-uniformity, gray level non-uniformity normalized, size-zone non-uniformity, size-zone non-uniformity normalized, zone percentage, gray level variance, zone variance, zone entropy, low gray level zone emphasis, high gray level zone emphasis, small area low gray level emphasis, small area high gray level emphasis, large area low gray level emphasis, large area high gray level emphasis |
| NGTDM (n = 5) | Coarseness, complexity, strength, contrast, busyness |

GLCM, gray level co-occurrence matrix; GLRLM, gray level run-length matrix; GLSZM, gray level size zone matrix; NGTDM, neighbouring gray tone difference matrix

The details of feature calculation are described at the Pyradiomics Site (https://pyradiomics.readthedocs.io/en/latest/features.html).

Supplementary Table 2. The baseline characteristics and neuropsychological test results in the TLE patients and HCs.

|  | TLE (n = 66) | HC (n = 65) | *P* value* |
| --- | --- | --- | --- |
| Age (years) | 42.5 ± 12.1 | 40.5 ± 12.1 | 0.409 |
| Sex |  |  | 0.138 |
| Female | 39 (56.5) | 30 (43.5) |  |
| Male | 27 (43.5) | 35 (56.5) |  |
| TLE lateralization |  |  | na |
| Right | 35 (53.0) |  |  |
| Left | 31 (47.0) |  |  |
| MMSE | 28.3 ± 1.7 | - | na |
| Neuropsychological data |  |  |  |
| Language and related function |  |  |  |
| K-BNT | 45.8 ± 34.8 | 81.4 ± 19.7 | < 0.001 |
| Verbal memory function (CVLT) |  |  |  |
| Immediate recall | 41.8 ± 28.8 | 53.8 ± 30.2 | 0.079 |
| Delayed recall | 40.0 ± 28.6 | 59.6 ± 28.0 | 0.004 |
| Recognition | 49.0 ± 25.9 | 56.7 ± 25.5 | 0.202 |
| Total | 52.3 ± 27.5 | 69.6 ± 21.4 | 0.005 |
| Visual memory function (RCFT) |  |  |  |
| Immediate recall | 40.3 ± 34.8 | 63.7 ± 35.3 | 0.004 |
| Delayed recall | 37.7 ± 33.8 | 62.4 ± 34.0 | 0.002 |
| Recognition | 27.7 ± 28.7 | 36.6 ± 28.3 | 0.185 |
| Frontal/ executive function |  |  |  |
| COWAT | 31.2 ± 29.7 | 56.5 ± 33.3 | 0.001 |
| Stroop test 1st | 75.6 ± 27.4 | 89.6 ± 11.5 | 0.001 |
| Stroop test 2nd | 80.1 ± 21.2 | 95.6 ± 7.2 | < 0.001 |
| Stroop test 3rd | 78.6 ± 25.5 | 95.0 ± 5.9 | < 0.001 |

Unless otherwise indicated, data are presented as number of patients (%).

^*^ Calculated from Student t test or Mann-Whitney test for continuous variables and chi-square test for categorical variables, to compare the characteristics of the TLE patients and HCs.

MMSE = Mini-Mental State Examination; K-BNT = Korean version of the Boston Naming Test, RCFT = Rey Complex Figure Test, CVLT = California Verbal Learning Test

Supplementary Table 3. Neuropsychological test results in the right and left TLE patients.

|  | Right TLE (n = 35) | Left TLE (n= 31) | *P* value* |
| --- | --- | --- | --- |
| MMSE | 28.3 ± 1.7 | 27.5 ± 5.0 | 0.392 |
| Neuropsychological data |  |  |  |
| Language and related function |  |  |  |
| K-BNT | 58.0 ± 32.1 | 32.9 ± 30.6 | 0.002 |
| Verbal memory function (CVLT) |  |  |  |
| Immediate recall | 46.4 ± 26.0 | 36.8 ± 31.2 | 0.179 |
| Delayed recall | 40.5 ± 5.7 | 39.4 ± 31.3 | 0.884 |
| Recognition | 50.5 ± 24.4 | 47.4 ± 27.8 | 0.637 |
| Total | 56.6 ± 23.6 | 47.6 ± 30.8 | 0.189 |
| Visual memory function (RCFT) |  |  |  |
| Immediate recall | 40.4 ± 33.7 | 40.1 ± 36.6 | 0.975 |
| Delayed recall | 38.0 ± 32.4 | 37.3 ± 35.8 | 0.930 |
| Recognition | 29.5 ± 29.5 | 25.8 ± 28.1 | 0.614 |
| Frontal/ executive function |  |  |  |
| COWAT | 30.2 ± 28.4 | 32.1 ± 30.3 | 0.924 |
| Stroop test 1st | 78.7 ± 25.4 | 72.1 ± 29.4 | 0.337 |
| Stroop test 2nd | 81.7 ± 19.2 | 79.2 ± 23.5 | 0.641 |
| Stroop test 3rd | 81.7 ± 23.1 | 75.2 ± 27.8 | 0.312 |

Unless otherwise indicated, data are presented as number of patients (%).

^*^ Calculated from Student t test or Mann-Whitney test for continuous variables and chi-square test for categorical variables, to compare the characteristics of the TLE patients and HCs.

CVLT = California Verbal Learning Test, K-BNT = Korean version of the Boston Naming Test, MMSE = Mini-Mental State Examination, RCFT = Rey Complex Figure Test

Supplementary Table 4. List of significant radiomics features to differentiate TLE from HC.

| Laterality | Feature category | Feature name |
| --- | --- | --- |
| right | first-order | kurtosis |
| right | first-order | skewness |
| right | GLCM | correlation |
| right | GLCM | informal measure of correlation 2 |
| right | GLRLM | run variance |
| right | GLSZM | gray level non-uniformity normalized |
| right | GLSZM | large area low gray level emphasis |
| right | GLSZM | small area low gray level emphasis |
| right | GLSZM | zone entropy |
| right | GLSZM | zone percentage |
| left | GLCM | informal measure of correlation 1 |
| left | GLSZM | large area emphasis |
| left | shape | maxium 2D diameter (column) |
| left | shape | maxium 2D diameter (row) |
| left | shape | maxium 2D diameter (slice) |
| left | shape | mesh volume |

GLCM = gray level co-occurrence matrix, GLRLM = gray level run-length matrix, GLSZM = gray level size zone matrix, GLDM = gray level dependence matrix, HC = healthy control, TLE = temporal lobe epilepsy

**References**

1 Kim, J. K. & Kang, Y. Normative study of the Korean-California Verbal Learning Test (K-CVLT). *Clin Neuropsychol* **13**, 365-369, doi:10.1076/clin.13.3.365.1740 (1999).

2 Shin, M.-S., Park, S.-Y., Park, S.-R., Seol, S.-H. & Kwon, J. S. Clinical and empirical applications of the Rey–Osterrieth complex figure test. *Nature protocols* **1**, 892 (2006).

3 Kim, H. & Na, D. L. brief report normative data on the Korean version of the Boston naming test. *J Clin Exp Neuropsychol* **21**, 127-133 (1999).
